# Exophthalmic Goitre and Its Relation to Diseases of the Thyroid Gland

**Published:** 1894-06-02

**Authors:** A. Maude


### EXOPHTHALMIC GOITRE, AND ITS RELATION TO DISEASE OF THE THYROID GLAND.—(*continued*).

By A. Maude.

We find Graves' disease associated by heredity with other nervous diseases; in fact, it is rare not to find a bad family history in this respect if the facts can be ascertained. A patient of the writer has one sister hystero-epileptic, and two others markedly hysterical; another patient's mother is myxœdematous, one uncle was insane; the mother's first cousin was imbecile, and had an imbecile child, while a brother of the patient is goitrous. Insanity, epilepsy, chorea, and drunkenness are all frequently found in the relatives of these sufferers. Another condition found both in relatives and in the patients themselves is diabetes. Acute rheumatism is also frequently a concomitant. But valvular disease of the heart is by no means common, though the heart, perhaps as the result of continued nervous irregularity, or perhaps from the action of some special toxin, or poison formed somewhere within the body, tends in most old-standing cases to become dilated and degenerate in structure. And it is right to insist that this degeneration and dilatation may—and sometimes does—cause sudden death. These changes in the heart may produce dropsy, chiefly in the legs, but tending in some instances to become as general and as serious as the dropsy produced by organic disease of the mitral valves, being, in fact, the result of imperfect action of the mitral valves.

There are other forms of dropsy, limited in many cases to small areas, such as the ankles, eyelids, or even single limbs, which are of nervous origin entirely; vasomotor changes, like the flushing and perspirations we noted above. These are, of course, of not such serious import, and are found to accompany many other nervous states.

8. The temperature also presents great changes, being very variable and frequently high. So constant is this fever sometimes that cases have been, when diarrhœa was also present, mistaken for typhoid fever. The writer has known of one instance personally, and the error has also been made by distinguished French physicians—*e.g.*, Peter.

There are numerous other small symptons, also very common, into which it is impossible in this space to enter fully; headache, giddiness, singing in the ears, a diminished resistance to electrical currents all over the body, are among them.

9. Another group of symptons, on which I laid great stress at the Medical Society, seem to point directly to "peripheral neuritis." Pain affecting the distribution of various sensory nerves, frequent cramp (resembling in the severe cases ordinary tetany), a constant diminution of the patellar reflex, and the very marked weakness of the legs, are, when grouped together, good evidence of some change in peripheral nerves. We now, from Professor Greenfield, learn that degenerative changes are always found in the great nerve trunks, particularly in the sciatic nerves, but the observations on this point are as yet only few.

10. The effect of partial removal of the thyroid gland in Graves' disease is very striking. Two years ago I collected about twenty cases in which this operation had been performed, and the improvement in a large number was obvious. Quite recently over forty cases have been recorded in an American paper. Any operation of magnitude is clearly dangerous in such patients, and not to be lightly undertaken. Attention is drawn to such interference solely as affording evidence on the question of pathology.

This incomplete clinical sketch suffices to show how widespread are the changes in the nervous system, and reflection on this fact must modify existing theories on its pathology. For many years it was accepted in England that the whole disturbance was due to irritation of the sympathetic, and all our leading text-books lay this down. Pathologists even succeeded, as they thought, in finding changes in the sympathetic ganglia present in a large proportion of post-mortem examinations. Then Dr. Hale White proved that these supposed changes were often present in other individuals, and could in no way be connected with exophthalmic goitre. Again, he showed that in some instances changes, such as small hæmorrhages, are to be found in the floor of the fourth ventricles, and accordingly the supposed seat of lesion was shifted to the medulla and the origins of the pneumogastric nerves.

Meanwhile in Germany Möbius, Müller, and Wette have been urging for some years that the increased and altered secretion of the thyroid plays an important part in the production of this group of symptoms. They are supported in this country by Dr. Byrom Bramwell, on clinical grounds by Dr. George Murray, and now by Professor Greenfield. The data on which the argument is based are briefly:—

*a*.—The comparison between the effects of thyroid feeding and the symptoms of Graves' disease.

*b*.—The contrast between the phenomena of myxœdema and Graves' disease. In one the atrophy of the thyroid, the slow pulse, the diminished perspiration, the subnormal temperature, the apathetic mental condition; in the other the direct opposite to all these.

*c*.—The effects of operation.

*d*.—The investigations of Dr. Greenfield show that in all cases there is a condition of the thyroid gland similar to the catarrh of other organs. The ordinary colloid contents of the vesicles of the gland become diminished, while a great proliferation of the epithelial cells lining those vesicles takes place. The variations in the gland, which I pointed out, are probably due (so Dr. Murray thinks, and with justice) to a turgescence similar to that which we know takes place in other glands, such as the salivary glands, during active secretion.

The inference (we cannot yet call it a conclusion) to be drawn is that the whole condition is due to some toxic material produced in the thyroid which affects the whole nervous system as much as lead or alcohol does, or the toxins produced in tetanus, hydrophobia, and beri-beri.

*A Short Bibliography of Some Recent English Papers.*—Maude, "Œdema in Graves' Disease," *Practitioner*,

Dec., 1891. Maude, "Gastric Crises in Graves' Disease," *Practitioner*, Oct., 1891. Maude, "Tremor in Graves' Disease," Brain lix. Murray, *Lancet*, Nov. 11, 1893. Greenfield, *Edinburgh Med. Journ.*, May, 1893. Greenfield, *Lancet*, Dec. 10, 1893 (Bradshaw Lectures); *British Med. Journal*, Dec. 10, 1893. Victor Horsley, Brown Lectures on the Thyroid, 1885. B. Bramwell, *Edinburgh Med. Journ.*, May, 1893.

